# Reinventing the evaluation wheel: COGwheel's co-designed digital innovation using the Qualtrics heat map

**DOI:** 10.1016/j.mex.2024.103147

**Published:** 2024-12-28

**Authors:** Mel Giugni, Alyssa R. Morse, Scott J. Fitzpatrick, Heather Lamb, Amelia Gulliver, Alison L. Calear, Vida Bliokas, Fiona Shand, Cassandra Chakouch, Bronwen Edwards, Louise A. Ellis, Purity Goj, Melissa Lee, Merkitta Main, Benn Miller, Glenda Webb, Ginny Sargent, Helen Skeat, Kelly Stewart, Kelly Wells, Carolyn McKay, Stride Safe Space Team, Erin Stewart, Michelle Banfield

**Affiliations:** a Centre for Mental Health Research, The Australian National University, Canberra, Australia; b School of Psychology, University of Wollongong, Wollongong, Australia; c Black Dog Institute, University of New South Wales, Sydney, Australia; d Roses in the Ocean, Brisbane, Australia; e Australian Institute of Health Innovation, Macquarie University, Sydney, Australia; f ACT Health Directorate, Canberra, Australia; g South Western Sydney Local Health District, Sydney, Australia; h Towards Zero Suicides Initiatives, South Western Sydney Local Health District, Sydney, Australia; i Population Health Exchange, The Australian National University, Australian Capital Territory, Australia; j Sonder, Adelaide, Australia; k Adelaide Primary Health Network, Adelaide, Australia; l Stride, Sydney, Australia (For details of the Stride Safe Space Team, visit https://stride.com.au); m Independent Consultant, Canberra, Australia; n The ALIVE National Centre for Mental Health Research Translation, The Australian National University, Canberra, Australia

**Keywords:** Co-design, Co-evaluation, Emotional distress, Suicide, Evaluation wheel, Strength-based assessment, Consumer experience, Outcome measure, COGwheel – Co-designed Outcomes for Guests Evaluation Wheel

## Abstract

The involvement of service–users, clinicians, and other health service end–users is recognised as an essential part of health and medical research. This collaborative approach can significantly contribute to methodological advancements including the development of research instruments and measures that ensure their suitability for research participants. The current paper details the co-design, development and implementation of the novel, digitised COGwheel (Co-designed Outcomes for Guests Evaluation Wheel). COGwheel is a brief, user-friendly digital instrument based on the Evaluation Wheel. It uses Qualtrics’ heatmap function to maximise ease of use for participants, alongside secure data collection, ease of analysis and simple interpretation. COGwheel was developed to evaluate peer-led, non-clinical ‘safe spaces’ for people experiencing emotional distress and/or suicidal crisis. However, the model has broader potential to be applied to other types of measures where it is essential to prioritise participant accessibility, comprehension, and ease of use.

• COGwheel is a brief, user-friendly digital instrument based on the Evaluation Wheel (Evaluation Support Scotland), that was co-designed by people with lived experience of suicidal crisis or distress.
• COGwheel addresses a significant gap in current evaluation methods, providing a novel digital instrument that also enhances accessibility and relevance for both participants and researchers.
• Utilising Qualtrics’ heatmap function, COGwheel enhances meaningful participant engagement and streamlines data collection, ensuring data security, interpretability, and efficient analysis.

## Graphical abstract

**Figure:**
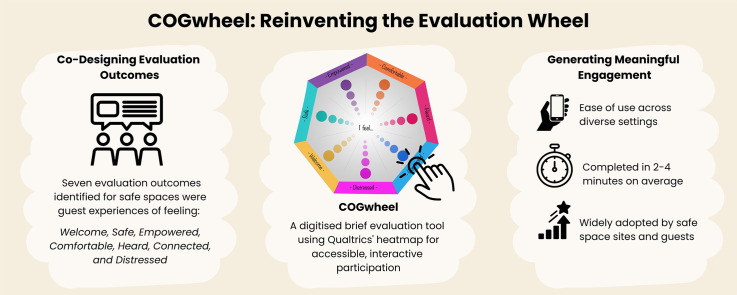

Specifications table

**Table:** 

Subject area:	Psychology
More specific subject area:	Health Psychology; Service Evaluation; Measurement/Scale; Evaluation methods & tools
Name of your method:	COGwheel – Co-designed Outcomes for Guests Evaluation Wheel
Name and reference of original method:	[1] ESS Evaluation Method: Evaluation Wheel, Evaluation Support Scotland. (2020). https://evaluationsupportscotland.org.uk/resources/evaluation-wheel/ (accessed November 14, 2023).
Resource availability:	COGwheel graphic available from: https://anu.au1.qualtrics.com/ControlPanel/Graphic.php?IM=IM_56X6FTlg3U7X6nQ

## Background

Research co-design is the meaningful engagement of end-users in the research process to ensure alignment between the aims and outcomes of research for all stakeholders [2]. Co-design has been recognised as best-practice in mental health service research in countries such as Australia and the UK [3,4]. Benefits of co-design include the relevance and appropriateness of data collection instruments, which in turn can help improve response rate and quality [5].

The ‘Co-Creating Safe Spaces' research project aimed to evaluate the feasibility and effectiveness of peer-led, non-clinical alternatives to the emergency department for people experiencing emotional distress and/or suicidal crisis [6]. This mixed methods, co-designed study involved a core team of academic researchers, including those with lived experience of suicidal crisis or distress, as well as health and community service managers, peer workers, and lived experience advocates.

Co-design began during the initial stages of the project, with service partners and lived-experience partners contributing to the formulation of research questions, the writing and reviewing of the funding application, and the development of the co-design approach. A co-design workshop in November 2021 brought together the core project team to discuss suitable outcome measures and data collection instruments for the evaluation.

Attendees emphasised the need for brief, engaging, and user-friendly measures of guest satisfaction that minimised participant burden. While some safe spaces used clinical measures of emotional distress, there was a clear preference for non-clinical, positive, and strengths-based measures.

This co-design workshop and further consultation with Stride staff at their symposium informed the development of a survey, which in December 2021 was distributed to 16 site partners and consumer representatives from the core project team. The objective was to gather further information on outcome measures from relevant personnel across partners involved in the delivery and evaluation of safe spaces, and to gauge interest on the most suitable methods for collecting these data within the contexts of routine practice. To identify the primary outcomes of interest, respondents were asked to suggest between three and five outcomes for guests of the safe space site/s they represented. Survey responses were received from 14 representatives across five safe space sites (Canberra, Blacktown, Wollongong, Northern Adelaide, and South Western Sydney), nominating primarily positive outcomes related to feelings of connection, safety, being listened to, and accepted. The final seven outcomes chosen for inclusion in the measure were: welcome, safe, empowered, comfortable, heard, connected, distressed. Although a contrast to the overall positive valence of the outcomes, we believed it was important to include “distress” in the measure, as this was a primary outcome for change across the study [6]. A review of existing validated measures failed to identify anything fit for purpose; that is, covering the key outcomes in an appealing and simple-to-use format for evaluation in non-clinical spaces. As such, we proceeded to develop a bespoke outcome measure for the Co-Creating Safe Spaces project.

## Method details

The COGwheel (Co-designed Outcomes for Guests Evaluation Wheel) was originally conceived as a paper-based tool inspired by the evaluation wheel template provided by Evaluation Support Scotland [1]. The evaluation wheel template offered a versatile tool for gathering information on nominated outcomes in a user-friendly manner. Respondents engage with an A4 printed wheel segmented by ‘spokes’ that represent different outcomes. They rate their experience by marking spokes or colouring segments to indicate their level of agreement. The COGwheel represents a seven-item questionnaire with Likert-type rating scales in a much more accessible format than a traditional survey.

However, ongoing discussions within the project team highlighted several challenges associated with the paper-based instrument, particularly in a formal research project. A key challenge related to difficulties in obtaining informed consent, specifically in effectively attaching the participant information sheet to a paper-based instrument and ensuring participants’ informed consent. This approach would place an undue burden on safe space staff by assigning them the ethical responsibility of obtaining informed consent. In addition, this introduced further logistical responsibilities around monitoring adequate supplies of the paper-based survey and ensuring the secure handling of data until collection by the research team — another important consideration for safeguarding participants' privacy and maintaining ethical standards – particularly as the sites were geographically dispersed across Australia. The need for subjective interpretation and manual data entry posed further potential challenges to maintaining scientific rigor and was expected to be labour-intensive. In response to these identified challenges, we initiated the development of a digitised evaluation wheel. Through multiple discussions and experimentation with various features within Qualtrics, we determined the ‘Heatmap’ function was a feasible option to support the digitised COGwheel.

**Fig. 1:**
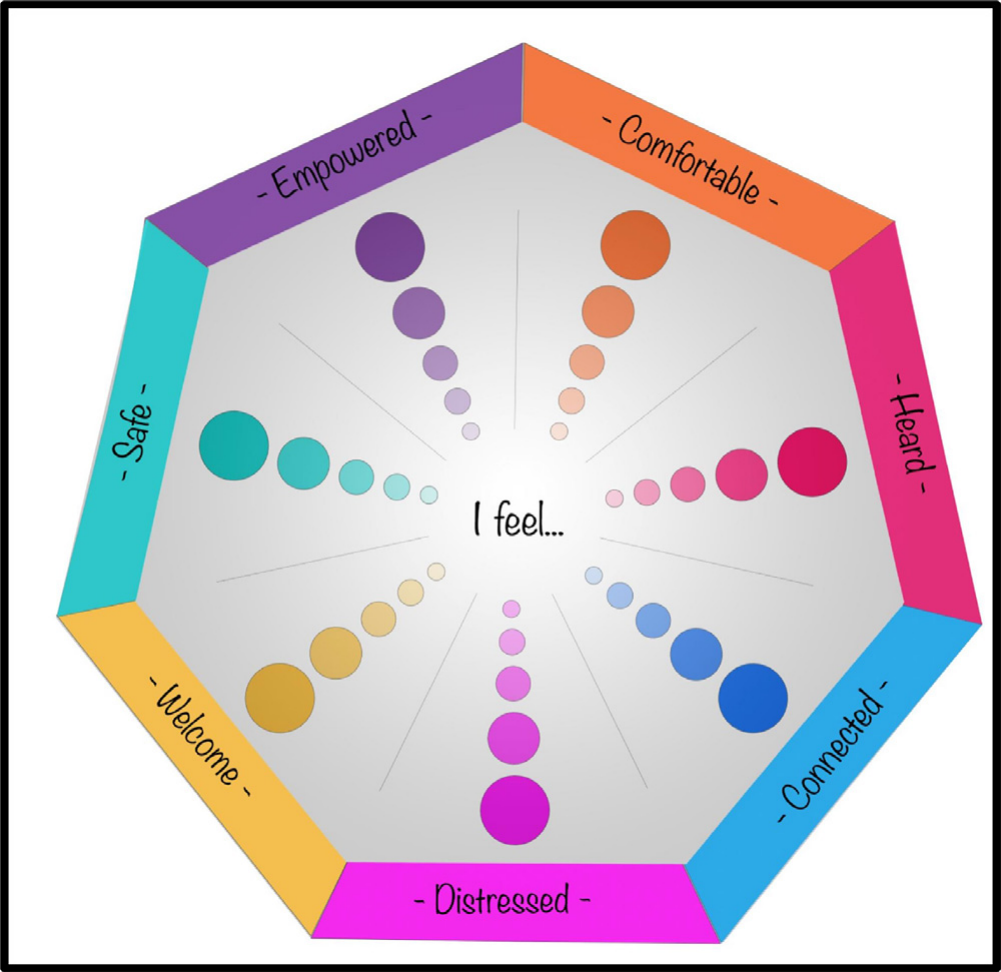
COGwheel graphic.

The technical development of the COGwheel commenced with the design of its visual elements. Using Adobe Photoshop CC 2019, the lead author (MG) created a visually appealing design focusing on user-friendliness. The goal was to craft a distinctive, interactive, and engaging experience, deliberately departing from visual themes associated with conventional clinical and research contexts. The graphic underwent multiple iterations, refined through ongoing discussions with the project team and trials on different platforms (e.g., screen sizes and settings, mouse input, touch screen input) to ensure usability and effectiveness. Fig. 1 displays the resulting image, which takes the form of a segmented heptagon. At its centre, the text reads “I feel…” serving as the anchor for seven sets of five coloured dots radiating outward. Each set aligns with a coloured heading, representing one of the seven co-designed guest outcomes. Increasing dot size represents increasing strength of feeling. The colour-coding scheme was designed to enhance clarity for participants by providing a distinct contrast between each segment of the heptagon. Additionally, the warm and inviting colour palette was chosen for its visual appeal, contributing to a more welcoming user experience. Once finalised, the image was saved as a JPEG file and uploaded to our Qualtrics project library.

**Fig. 2:**
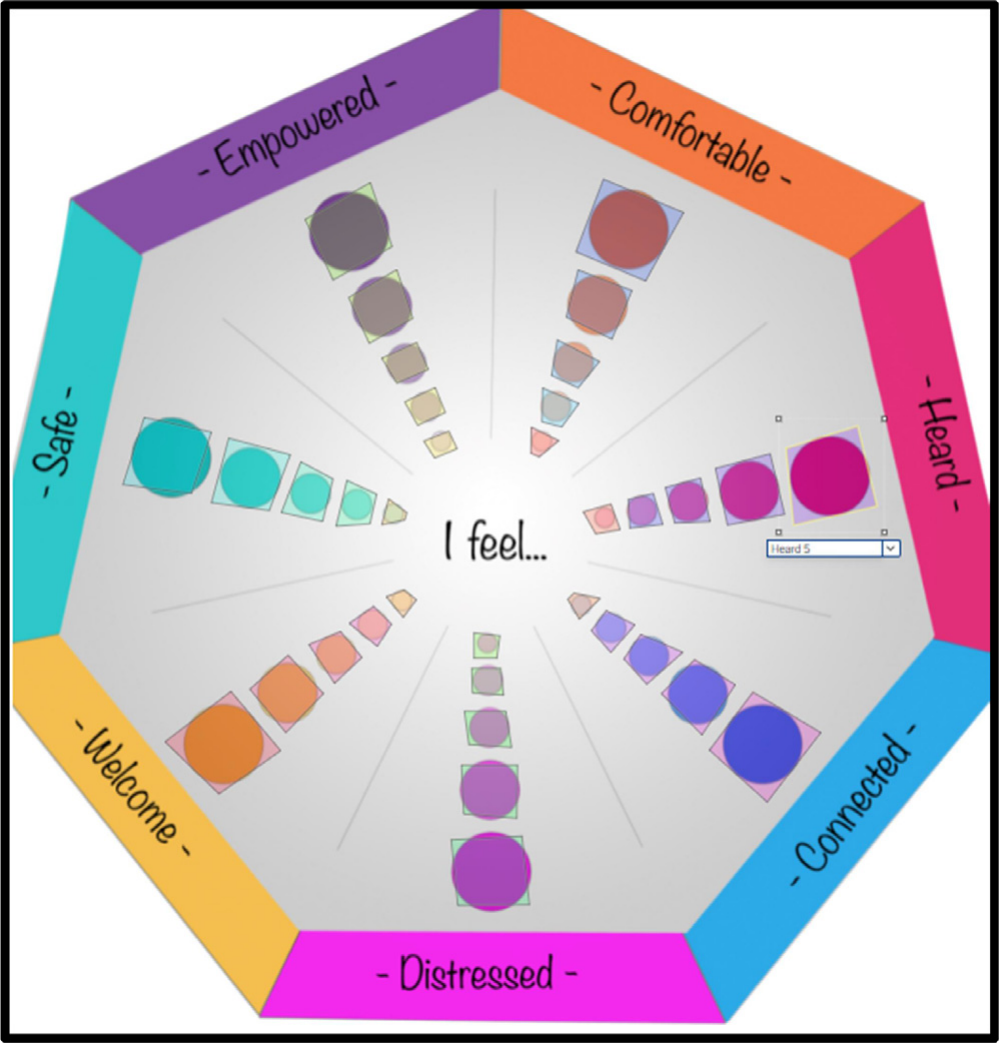
Labelled heatmap regions applied to COGwheel graphic.

Survey construction began with a blank survey, incorporating the participant information sheet and informed consent questions in the initial block. Participants were required to acknowledge reading the information sheet and explicitly consent to participate in the research before proceeding. The heatmap question was added to the subsequent block, where we integrated our COGwheel graphic/image from the project library. To reduce ambiguity in data interpretation, we utilised the ‘add region’ feature to define discrete areas within the image, pictured in Fig. 2. This approach simplifies reporting by quantifying clicks in predefined regions, as opposed to the default setting that only reports the coordinates for each click. Within each segment, discrete regions were applied to each of the 5 circles, labelled with the segment heading as a prefix followed by a number from 1 to 5. For example, in the `comfortable' segment, the smallest inner circle was labelled `Comfortable 1', gradually increasing in size to the largest and outermost circle ‘Comfortable 5’. This labelling mirrors a Likert scale response where 1 signifies the lowest possible score, and 5 indicates the highest possible score. By creating and labelling regions in this manner, we automated and standardised the interpretation of response data, offering similar benefits to those of a traditional online Likert-type survey.

It is important to note that regions and their labels were not visible to respondents, therefore instructions for respondents had to be quite clear: “Using the evaluation wheel below, click/tap on a circle under each heading to express how you felt during your visit to (Safe Space / Safe Haven). (The smallest circle represents “not at all”, whereas the largest circle represents “very much”)”.

**Fig. 3:**
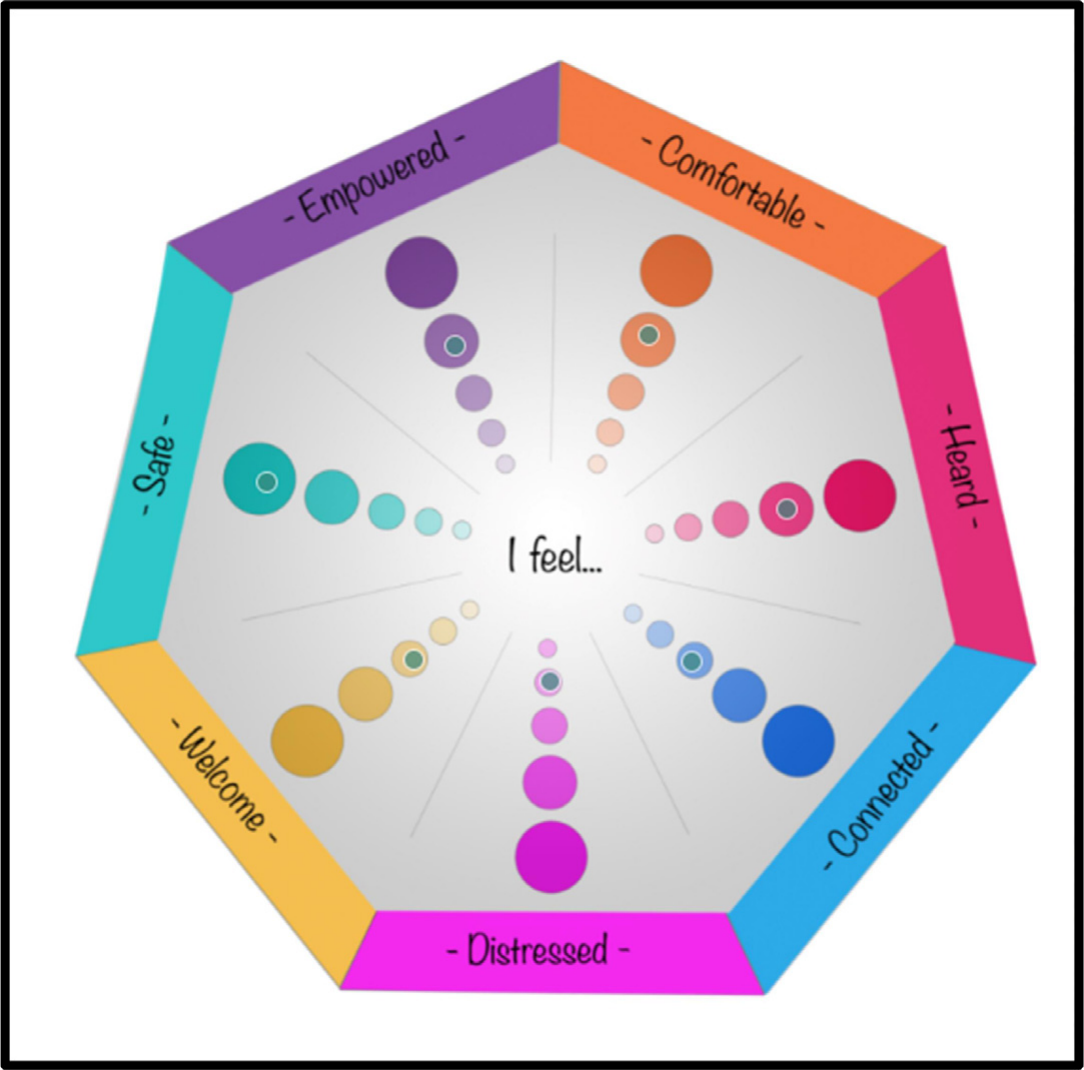
Respondent view of click selections on COGwheel.

When respondents clicked on the image, a small dot appeared on the screen to indicate exactly where was clicked, as displayed in Fig. 3. Respondents could modify their choices by clicking on the small dot to remove it or by clicking and dragging it to a new location. To help ensure only one selection was made per segment, the ‘Interaction’ settings in the heatmap question type of Qualtrics were configured to limit the total number of clicks on the graphic to seven. Similarly, the 'Response Requirement' settings in Qualtrics were configured with custom validation rules (provided in the supplementary material) to alert respondents if they missed a segment or if one or more of their selections were not captured within the predefined regions. If this occurred, respondents were presented with a custom error message: 'Please ensure one dot has been placed in each section (one under each heading). If you continue to get this error message, you may need to move the dots slightly so that they are centred within the selected circle.' This message provided clear instructions for resolving common issues and ensuring data accuracy. Once respondents were satisfied with their selections, they clicked ‘Next' to proceed with submission of their responses.

### Data visualisation and analysis

**Fig. 4:**
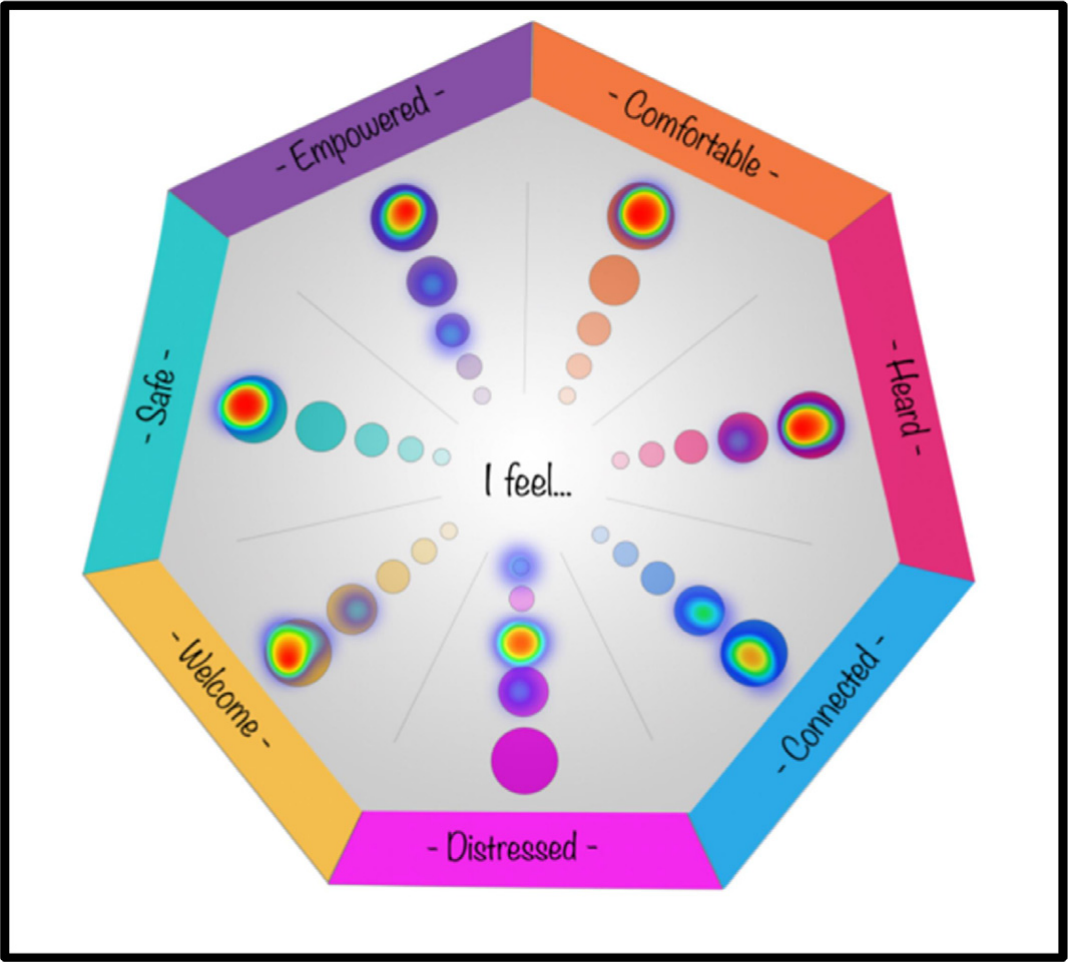
Heat map plot of COGwheel responses.

For a quick and intuitive display of response distributions across the guest outcomes, data are visually represented through a ‘heat map plot’, accessible via the ‘Results’ tab in Qualtrics. Fig. 4 provides an example heat map plot.

Analysis of the collected data was primarily conducted using SPSS software. Syntax provided in the supplementary material, was designed to compute new variables for each guest outcome, labelled ‘EmpowermentScore’, ‘ComfortableScore’, ‘DistressScore’, ‘SafeScore’, ‘HeardScore’, ‘ConnectedScore’, and ‘WelcomeScore’. These values were assigned numerical values between one to five, based on the regions respondents selected within the respective segments of the COGwheel. To illustrate, if a respondent clicked on the largest outermost circle under the heading of ‘Empowerment’, this resulted in a computed score of five on the ‘EmpowermentScore’ variable.

The derived outcome scores serve to provide quantification of participants’ subjective experience during a visit to the safe space, encompassing the seven co-designed guest outcomes. A score of five means they felt strongly (e.g. felt very safe or felt very distressed), and a score of one means they did not experience the feeling at all (e.g., felt not at all safe or felt not at all distressed).

### Challenges encountered

The digitisation of the COGwheel introduced various limitations despite our efforts to ensure user-friendliness. Creating this innovative instrument using technology not originally intended for its purpose presented its own set of challenges that may have impacted respondent experiences. These challenges, although anticipated and addressed as far as possible, highlight areas for potential improvement and refinement in future iterations of the instrument.

Traditional Likert-type scale measures within Qualtrics can be easily limited to single responses; however, our use of the heatmap presented a unique challenge as all seven scales were embedded within one graphic. To address this, we limited the total number of clicks to seven and implemented validation to ensure that at least one region within each segment was clicked upon. However, rather than preventing respondents from adding new selections, limiting the clicks to seven resulted in the newest click replacing the oldest one after seven click selections. This functionality may have created confusion for respondents as their earlier selections unexpectedly shifted or disappeared after reaching the seven-click limit. When the COGwheel is used in services or in-person research contexts, providing a demonstration to first-time users may overcome this technical limitation.

Guests were prompted to make selections in all segments to submit their response, deviating from the flexibility allowed in our other surveys where guests could choose to answer and submit only what they felt comfortable with. In future iterations, the inclusion of a “Not Applicable’ or ‘Unsure’ option in each segment should be considered to enhance respondent autonomy.

Additionally, a challenge emerged regarding the interpretation of the distress variable as the only negative outcome. The tool was purposely designed for all Likert-type scales to operate in the same direction, i.e., higher score equal to more of the outcome. However, unlike the other six outcomes where a higher score was desirable, a higher score on distress represented a poorer outcome. This ambiguity may have led to varying respondent interpretations. However, the use of a mixture of positive and negatively worded items to reduce response bias and increase engagement is common [7], so the impact may not be meaningful when compared with other ways of presenting the items such as in traditional Likert-type scales. In future validations, this potential limitation could be addressed by identifying how respondents typically interpret the COGwheel, using methods such as cognitive interviewing.

One of the main challenges encountered during the transition to a digitised version was the sizing of the COGwheel graphic. To create a tool suitable for mobile devices, the graphic size was reduced to accommodate smaller screens. However, this resulted in the dimensions of the heatmap regions and their spacing becoming too small for users to accurately select the specific regions. As a compromise to maximise user-friendliness, an image size was selected that allowed for easier selection of the regions, though this necessitated horizontal scrolling on smartphone and smaller tablet screens to visualise the full image, which was less than ideal. Furthermore, some of the circles were still small enough that they may have been difficult or frustrating for respondents to select, especially those using a smartphone device. When data collection is conducted in-person, provision of standardised touchscreen devices could support consistent sizing and usability. In future, formal user experience testing could identify the most common challenges for smartphone users and potential strategies to overcome them.

### Advantages of the COGwheel

Despite the challenges of the digital platform, one of its key advantages is flexibility. The COGwheel can be implemented in a variety of ways, depending on the requirements and resources of the health service or research project. For example, in the Co-Creating Safe Spaces project, the COGwheel enabled the collection of consistent data across multiple service sites that delivered the tool in different ways, including the use of service-owned tablets, researcher-provided tablets and participants’ personal devices. Informed consent processes and instrument scoring are consistent across implementation options, and scoring is simple and efficient using the syntax provided. The COGwheel is also easy to disseminate as it can be accessed via an anonymous QR code or website link, both generated through the Qualtrics platform.

Through co-designing the COGwheel we aimed to create a measure of guest experiences that was brief and involved minimal participant burden. Meta-data and observations from our use of COGwheel suggest that the instrument has achieved this goal. In our study, based on 75 responses collected, participants typically completed the COGwheel within 2 to 4 min. The median completion time was 2 min and 10 s (IQR: 1 min and 23 s to 3 min and 46 s). The co-designed experience measures are also likely to be more engaging than standard satisfaction or distress surveys, as they provide participants with an opportunity to report on outcomes that are meaningful to people with lived experience of suicidal crisis or distress. A formal evaluation of user experiences is planned to support these conclusions and identify potential areas for improvement. This is particularly important if the COGwheel is implemented in new contexts, to confirm the instrument is user-friendly, relevant, and acceptable for use in different populations.

Regular meetings and interactions with staff members from our participating safe space sites have indicated that the COGwheel has been well received. Widespread adoption of the measure across multiple sites, and prolonged use over a period of several months, speaks to the engaging and accessible design of COGwheel. The COGwheel was the most popular evaluation tool developed for the Co-Creating Safe Spaces project, selected for implementation at all six sites and with the highest guest completions. Service staff implementing the COGwheel benefitted from completing a demonstration and practice session to familiarise themselves with the tool, but these were not time-consuming and were able to fit into regular team meetings. Stakeholders (including peer workers, other service staff members, and people with lived experience) also expressed appreciation of the positive language, visual appeal, and intuitive user-friendly interface. For example, one site requested the COGwheel image be placed on posters to encourage people to participate in the evaluation. These experiences suggest enthusiastic endorsement for the COGwheel and its departure from conventional survey instruments which may feel clinical or impersonal.

### Conclusion

The COGwheel is a brief, user-friendly digital instrument that facilitates the flexible and secure collection of guest data on service satisfaction and experience. The seven outcomes were co-designed by people with lived experience of suicidal crisis or distress, ensuring they are meaningful and relevant to people from this population. The novel visual presentation of the instrument is attractive to health service staff, including peer workers, facilitating its uptake and successful implementation. Implementation challenges remain, including improving user experiences on smartphones and validating users’ interpretation of outcome items. Future research that aims to address these challenges should actively involve people with lived experience in the research process, in keeping with the values driving the design of the COGwheel.

## Method validation

‘Not applicable’.

## Limitations

‘Not applicable’.

## Ethics statements

This research was approved by the Australian Capital Territory Health Human Research Ethics Committee (Reference Number 2022.ETH.00043). Informed consent was obtained from all participants prior to their involvement in the study.

## CRediT authorship contribution statement

**Mel Giugni:** Conceptualization, Methodology, Software, Data curation, Writing – original draft. **Alyssa R. Morse:** Conceptualization, Investigation, Writing – original draft, Writing – review & editing. **Scott J. Fitzpatrick:** Conceptualization, Writing – original draft, Writing – review & editing, Supervision, Project administration. **Heather Lamb:** Conceptualization, Writing – original draft, Writing – review & editing. **Amelia Gulliver:** Conceptualization, Writing – original draft, Writing – review & editing. **Alison L. Calear:** Conceptualization, Writing – review & editing. **Vida Bliokas:** Conceptualization, Writing – review & editing. **Fiona Shand:** Conceptualization, Writing – review & editing. **Cassandra Chakouch:** Conceptualization, Writing – review & editing. **Bronwen Edwards:** Conceptualization, Writing – review & editing. **Louise A. Ellis:** Conceptualization, Writing – review & editing. **Purity Goj:** Conceptualization, Writing – review & editing. **Melissa Lee:** Conceptualization, Writing – review & editing. **Merkitta Main:** Conceptualization, Writing – review & editing. **Benn Miller:** Conceptualization, Writing – review & editing. **Glenda Webb:** Conceptualization, Writing – review & editing. **Ginny Sargent:** Conceptualization, Writing – review & editing. **Helen Skeat:** Conceptualization, Writing – review & editing. **Kelly Stewart:** Conceptualization, Writing – review & editing. **Kelly Wells:** Conceptualization, Writing – review & editing. **Carolyn McKay:** Conceptualization, Writing – review & editing. **Stride Safe Space Team:** Conceptualization, Writing – review & editing. **Erin Stewart:** Conceptualization, Writing – review & editing. **Michelle Banfield:** Conceptualization, Methodology, Investigation, Writing – original draft, Writing – review & editing, Supervision, Project administration, Funding acquisition.

## Declaration of competing interest

The authors declare that they have no known competing financial interests or personal relationships that could have appeared to influence the work reported in this paper.

## Data Availability

No data was used for the research described in the article.

## Appendix Supplementary materials

Image, application 1

Image, application 2
