# Supplementary material for: Reinventing the evaluation wheel: COGwheel's co-designed digital innovation using the Qualtrics heat map
Source: MethodsX. 2024 Dec 28;14:103147. doi: 10.1016/j.mex.2024.103147 (PMC11751559; doi:10.1016/j.mex.2024.103147)
Supplement: Supplementary file 1 [file mmc1.docx]

Supplementary File 1: Screenshot of the custom validation settings for the heatmap question used in the survey design.

*
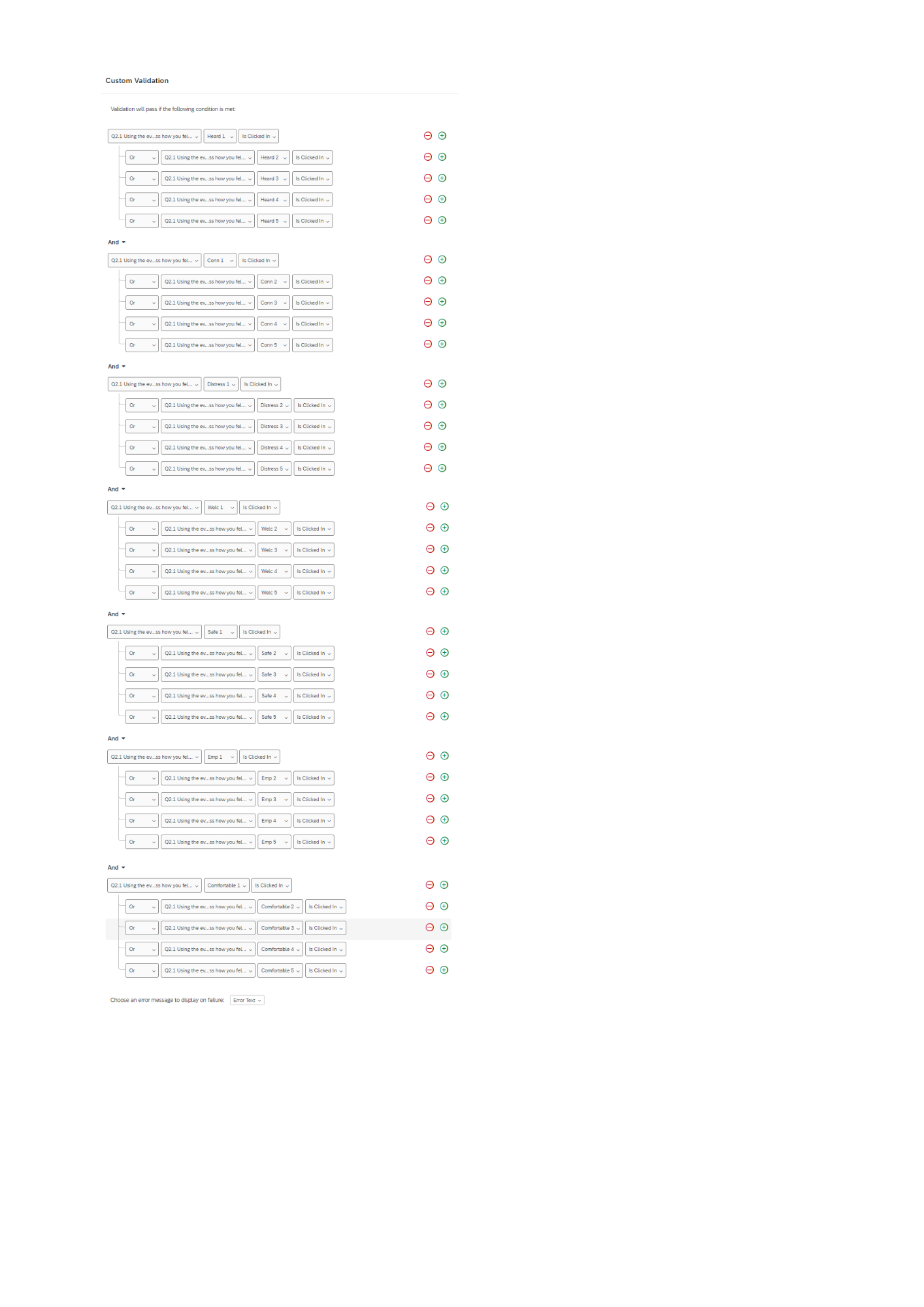
*
