# Supplementary material for: Reinventing the evaluation wheel: COGwheel's co-designed digital innovation using the Qualtrics heat map
Source: MethodsX. 2024 Dec 28;14:103147. doi: 10.1016/j.mex.2024.103147 (PMC11751559; doi:10.1016/j.mex.2024.103147)
Supplement: Supplementary file 2 [file mmc2.docx]

Supplementary File 2: SPSS syntax for creating guest outcome scores used in the evaluation of peer-led safe spaces.

COMPUTE EmpowermentScore = 0.

IF (Q2.1_Emp1 = 1) EmpowermentScore = 1.

IF (Q2.1_Emp2 = 1) EmpowermentScore = 2.

IF (Q2.1_Emp3 = 1) EmpowermentScore = 3.

IF (Q2.1_Emp4 = 1) EmpowermentScore = 4.

IF (Q2.1_Emp5= 1) EmpowermentScore = 5.

EXECUTE.

COMPUTE ComfortableScore = 0.

IF (Q2.1_Comfortable1= 1) ComfortableScore = 1.

IF (Q2.1_Comfortable2 = 1) ComfortableScore = 2.

IF (Q2.1_Comfortable3 = 1) ComfortableScore = 3.

IF (Q2.1_Comfortable4 = 1) ComfortableScore = 4.

IF (Q2.1_Comfortable5 = 1) ComfortableScore = 5.

EXECUTE.

COMPUTE DistressScore = 0.

IF (Q2.1_Distress1= 1) DistressScore = 1.

IF (Q2.1_Distress2 = 1) DistressScore = 2.

IF (Q2.1_Distress3 = 1) DistressScore = 3.

IF (Q2.1_Distress4 = 1) DistressScore = 4.

IF (Q2.1_Distress5 = 1) DistressScore = 5.

EXECUTE.

COMPUTE SafeScore = 0.

IF (Q2.1_Safe1= 1) SafeScore = 1.

IF (Q2.1_Safe2 = 1) SafeScore = 2.

IF (Q2.1_Safe3 = 1) SafeScore = 3.

IF (Q2.1_Safe4 = 1) SafeScore = 4.

IF (Q2.1_Safe5 = 1) SafeScore = 5.

EXECUTE.

COMPUTE HeardScore = 0.

IF (Q2.1_Heard1= 1) HeardScore = 1.

IF (Q2.1_Heard2 = 1) HeardScore= 2.

IF (Q2.1_Heard3 = 1) HeardScore = 3.

IF (Q2.1_Heard4 = 1) HeardScore = 4.

IF (Q2.1_Heard5 = 1) HeardScore = 5.

EXECUTE.

COMPUTE ConnectedScore = 0.

IF (Q2.1_Conn1= 1) ConnectedScore = 1.

IF (Q2.1_Conn2 = 1) ConnectedScore = 2.

IF (Q2.1_Conn3 = 1) ConnectedScore = 3.

IF (Q2.1_Conn4 = 1) ConnectedScore = 4.

IF (Q2.1_Conn5 = 1) ConnectedScore = 5.

EXECUTE.

COMPUTE WelcomeScore = 0.

IF (Q2.1_Welc1= 1) WelcomeScore= 1.

IF (Q2.1_Welc2 = 1) WelcomeScore = 2.

IF (Q2.1_Welc3 = 1) WelcomeScore = 3.

IF (Q2.1_Welc4 = 1) WelcomeScore = 4.

IF (Q2.1_Welc5= 1) WelcomeScore = 5.

EXECUTE.
